# Detecting hospital behaviors of up-coding on DRGs using Rasch model of continuous variables and online cloud computing in Taiwan

**DOI:** 10.1186/s12913-019-4417-2

**Published:** 2019-09-04

**Authors:** Tsair-Wei Chien, Yi-Lien Lee, Hsien-Yi Wang

**Affiliations:** 1 Medical Research Department, Chi Mei Medical Center, Tainan, Taiwan; 2 Department of Medical Affairs Chi Mei Medical Center, Tainan, Taiwan; 3 Institute of Information Management, National Chung Cheng University, Chiayi, Taiwan; 4 Department of Sport Management, College of Leisure and Recreation Management, Chia Nan University of Pharmacy and Science, Tainan, Taiwan; 5 NephrologyDepartment, Chi Mei Medical Center, 901 Chung Hwa Road, Yung Kung Dist., Tainan, 710 Taiwan

**Keywords:** Rasch analysis, Standardized residual analysis, Dashboard, Medical center

## Abstract

**Background:**

This work aims to apply data-detection algorithms to predict the possible deductions of reimbursement from Taiwan’s Bureau of National Health Insurance (BNHI), and to design an online dashboard to send alerts and reminders to physicians after completing their patient discharge summaries.

**Methods:**

Reimbursement data for discharged patients were extracted from a Taiwan medical center in 2016. Using the Rasch model of continuous variables, we applied standardized residual analyses to 20 sets of norm-referenced d**i**agnosis-related group (DRGs), each with 300 cases, and compared these to 194 cases with deducted records from the BNHI. We then examine whether the results of prediction using the Rasch model have a high probability associated with the deducted cases. Furthermore, an online dashboard was designed for use in the online monitoring of possible deductions on fee items in medical settings.

**Results:**

The results show that 1) the effects deducted by the NHRI can be predicted with an accuracy rate of 0.82 using the standardized residual approach of the Rasch model; 2) the accuracies for drug, medical material and examination fees are not associated among different years, and all of those areas under the ROC curve (AUC) are significantly greater than the randomized probability of 0.50; and 3) the online dashboard showing the possible deductions on fee items can be used by hospitals in the future.

**Conclusion:**

The DRG-based comparisons in the possible deductions on medical fees, along with the algorithm based on Rasch modeling, can be a complementary tool in upgrading the efficiency and accuracy in processing medical fee applications in the discernable future.

**Electronic supplementary material:**

The online version of this article (10.1186/s12913-019-4417-2) contains supplementary material, which is available to authorized users.

## Background

Fee-for-service (FFS) is a payment system, in which the health care providers are paid for each service performed [1]. To reduce the rapid growth rate of health expenditures, diagnosis-related groups (DRGs) are launched according to patients with similar clinical characteristics, resource consumption patterns, and comparable costs [2].

Taiwan’s National Health Insurance (NHI) scheme, launched in 1995, originally used FFS. Despite various legislative and administrative measures aimed at capping maximum reimbursements, including a global budget system and a case-payment scheme, the rapid increase of medical expenses continued to occur [3, 4]. In response, the Bureau of National Health Insurance (BNHI) began using a Taiwan-specific DRG system (TW-DRG) in January 2010, and a total of 1663 TW-DRGs were developed until 2016.

The main problem here is how to detect hospitals’ behaviors of up-coding on DRGs [5]. Traditionally, the BNHI adopts the peer-review approach by giving the whole list of medical expenditures (or say items) to physicians from other hospitals for examining whether the reimbursement case is rational and reasonable. An efficient and effective detection method can be thus expected to improve using item response theory (IRT) modeling, particularly the one-parameter Rasch model [6] of continuous items [7, 8]. Because that (1) such cases of inpatient expenditures are continuous variables, (2) IRT-bases Rasch model is one-parameter simple model requiring relatively small sample size to calibrate model parameters, and (3) the Rasch model of continuous items [7, 8] has been developed before in the literature, an online routine (or say application programming interface, API) can be used for detecting abnormal up-coding behaviors on DRGs [5]. Importantly, the detection should be (and must be) objective and scientifical as much as possible.

Given that the DRGs are characterized by similar resource consumption patterns and comparable costs [2], any up-coding in a discharge case results in items being miss-fitted to the model if the standardized residual analysis [9] is applied to inspect. That is, the Z-score on the response interacted by the momentum of the case and the item equals $\frac{\left( observed-\exp ected\right)}{SD}$, where SD = standard deviation on the item and the case [10, 11].

To solve the problem, two approaches are implemented in the current study: (1) verifying the effectiveness of the Rasch standardized residual analysis in terms of DRG detection, and (2) developing an online detection scheme for tracking any item misfitting to the model. The latter approach can help alert physicians once they accomplish their inpatient’s discharge summaries, allowing them to prepare the necessary notes (or actions) before the BNHI assessment on the reimbursement of medical expenditures has been implemented.

In this work, we aim to apply the Rasch model of continuous variables [7, 8] (1) to verify the effectiveness of detection on TW-DRGs, and (2) to develop an online checking tool for selecting the most misfit items on TW-DRGs for each inpatient case.

## Methods

### Data source

#### Experimental and control groups

We applied the TW-DRG classification module issued by the BNHI to two groups, namely, the control and experimental groups.

##### Control group

A set of 300 cases (as norm-reference) from 20 TW-DRGs(i.e., like types of tests) were randomly selected from a medical center in southern Taiwan between 2015 and 2016 and were not deducted yet by the BNHI assessment for the medical fees on any item. These 300 cases were used for calibrating item (or say fee) parameters(i.e., item difficulties on IRT terms) as references comparable to the experimental group.

##### Experimental group

We randomly selected 194 cases on the 20 TW-DRGs mentioned above from the studied medical center at the same period(i.e., 2015 and 2016). Data on these 194 cases were submitted to BNHI for reimbursement before and were already deducted by the BNHI assessment for the medical fees on at least one item.

#### Medical fees with continuous responses

Given that all items with continuous responses were appropriately applied to the algorithm of the Rasch model of continuous variables [7, 8], all those 17 medical fees on 20 TW-DRGs for 300 cases in the control group were included for calibrating the parameters of item difficulty. If no expenditure on any item existed, the missing data were considered accordingly.

**Fig. 1:**
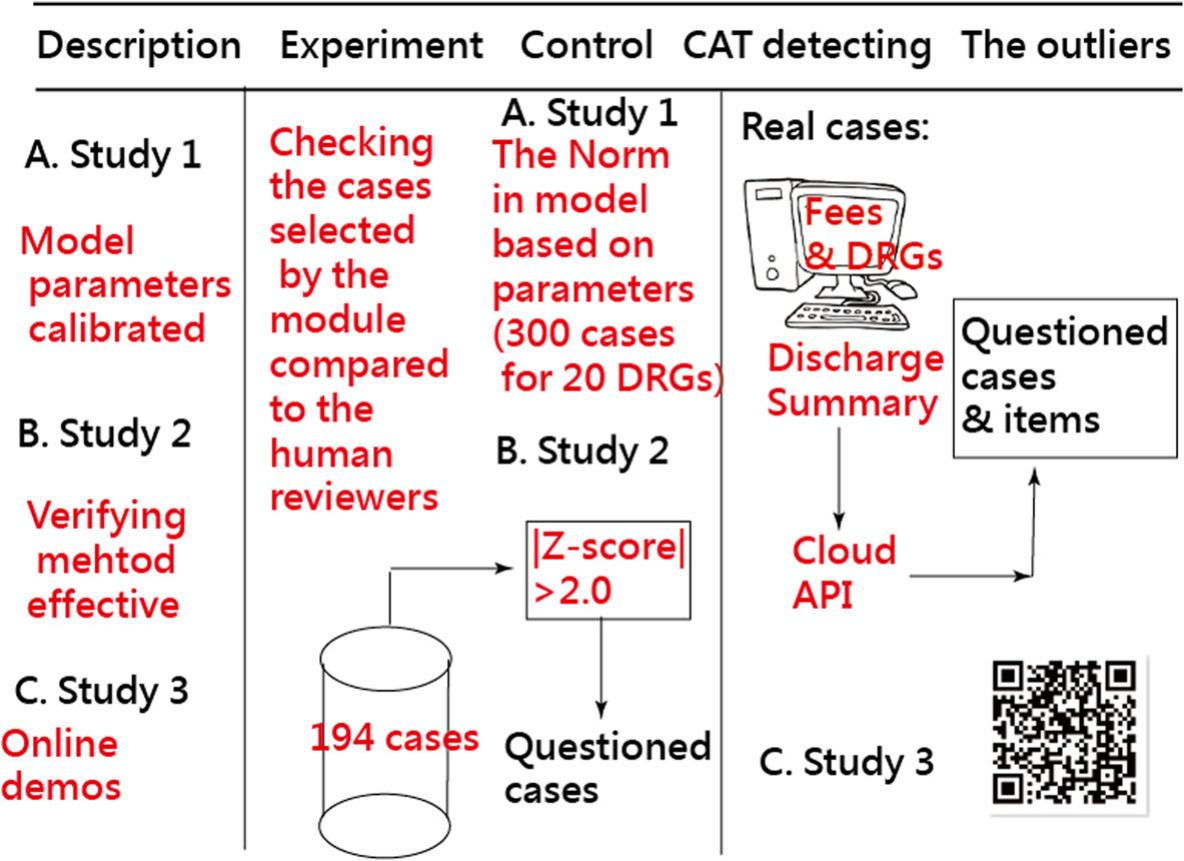
Study flowchart

The other 194 cases were examined using the computerized adaptive testing (CAT) technique [12–16] because not all cases were observed having these 17 medical fees in reimbursement. The aim is to determine whether the results (i.e., the outliers of the Z-score beyond 2.0 on responses) are similar to the detected items examined by the BNHI (see Fig. 1).

#### The mathematical form of the Rasch model for continuous item responses

The mathematical form of the Rasch model of continuous variables [7, 8] can be simply expressed by the equations:

1 $$ \mathrm{Probability}=\frac{\exp \left({\theta}_n-{\delta}_i\right)}{\left(1+\exp \left({\theta}_n-{\delta}_i\right)\right)}, $$

Where the response probability for a case performance (theta) on an item (delta) is shown in Eq. (1). Newton-Raphson iteration method can be applied to estimate case performances and item difficulties [7, 8, 17]. If item difficulties for a DRG have been known(i.e., Study A in Fig. 1), any case performance is estimated by Eq. (2).

2 $$ {\theta}_1={\theta}_0+\frac{\left({O}_{ni}-{E}_{ni}\right)}{Var_{ni}}={\theta}_0+\frac{\operatorname{Re}{sidual}_{ni}}{E_{ni}\times \left(1-{E}_{ni}\right)}, $$

The iteration process for estimating case performance is in Eq. (2). Through which, the latter theta is determined by the former theta and the residual divided by the variance. The expected value(*E*_*ni*_) for the case on an item is computed in terms of probability in Eq. (1). The observed scores(i.e., medical fees denoted by *O*_*ni*_ in Eq. (2)) has been transformed to a percentage ranging from 0 to 1.0 based on the maximum and the minimum in the target DRGs.

3 $$ \mathrm{The}\ \mathrm{Z}\hbox{-} \mathrm{score}\ \mathrm{for}\ \mathrm{the}\ \mathrm{case}\ \mathrm{on}\ \mathrm{an}\ \mathrm{item}=\frac{\left({O}_{ni}-{E}_{ni}\right)}{\sqrt{Var_{ni}}}, $$

Using the CAT process ignoring the item with zero(i.e., no expenditure deemed as missing data), case performance and Z-score are yielded by the Eqs. (2, 3).

#### Study targets and statistics

Three types of items (i.e., medical fees) were examined, namely, drugs, materials, and examinations, on 194 cases regarding TW-DRGs. Chi-square test was performed to determine whether the number of deducted items and the association between years (i.e., 2015 and 2016) was consistent.

The standardized residual Z-score(i.e., the continuous predicting variable) was used to investigate whether the accuracy on the binary variable (i.e., true and false on deduction) can be considered significant and acceptable using the Receiver Operating Characteristic (ROC) [18].

Furthermore, the 3298 cells of the Z-score in the experiment group (= 194 × 17) were compared and examined by the significant level beyond 2.0 [10–16] due to 1.96 standard deviation from the mean(i.e., zero) suitable for large sample and 2.0 for small sample when the probability of type I error less than 0.05.

### Data analysis

We assumed that all identical TW-DRGs were grouped in similar patterns across medical fees. Once any item showed an abnormal response (i.e., Z-score > 2.0, *p* < 0.05), this was highlighted and pegged as the case that may be possibly deducted by the BNHI in the future. The online module [19] on cloud computing will be performed and demonstrated in this study. Samples of research data were deposited in Additional file 1. The prototype of the demonstration program online was present in Additional files 2 and 3 with an MP4 video. The Excel module of the Rasch model for continuous item responses was shown in Additional file 4, which was extracted from the previous study [7, 8].

The correct rate computed in this study is applied by the equation (=correct number/total number in the experimental group).

### Ethical approval

All the data used in this study were extracted from a medical center; thus, we obtained ethical approval according to the regulation of the Taiwan Ministry of Health and Welfare. The document was coded (No. 10602-E03) and approved by the IRB of the Chi Mei Medical Center, Taiwan.

## Results

### Comparison of the deduction counts between 2015 and 2016

As all 194 cases have at least one item of the medical fee deducted by the BNHI, we applied the Z-score to examine the accuracy of the deduction made in the count. The correct prediction rate is 0.82, as shown in Table 1. No difference in count exists between 2015 and 2016 (*p* = 0.19), implying that around 18% of the cases are deducted with Z-score < 2.0. Those cases (i.e., Z < 2.0) may be the subject of debate between the BMHI and the hospital physicians. The three category targets(i.e., drugs, materials, and examinations) do not show any differences (*p* = 0.68, *p* = 0.52, *p* = 0.50) based on the Chi-square test results. Among them, the examination category presents the highest percentage (73%), followed by the material (66%) and the drug (61%) categories (Table 2).

**Table 1:** Total cases examined by count using Chi-square test

Year	Correct	Incorrect	Sum	*χ* ^2^	Rate
2004	154	40	194	0.19	0.80
2005	164	30	194		0.85
Mean	159	35	194		0.82

*p* = 0.19; correct rate = #correct/sum

**Table 2:** The three categories (drug, material, and examination) showed equal counts between 2015 and 2016

Year	Drug					Material					Examination				
	Corr.	Incorr.	Sum	*χ* ^2^	Rate	Corr.	Incorr.	Sum	*χ* ^2^	Rate	Corr.	Incorr.	Sum	*χ* ^2^	Rate
2015	120	74	194	0.68	0.62	132	62	194	0.52	0.68	138	56	194	0.50	0.71
2016	116	78	194		0.60	126	68	194		0.65	144	50	194		0.74
總和	236	152	388		0.61	258	130	388		0.66	282	106	388		0.73

*p* > 0.05; correct rate = #correct/sum

### ROC curve under the area

The ROC areas within 0.65 and 0.70 for the three categories in 2015 and 2016 present a significant difference (*p* < 0.05), as shown in Table 3. The 2 years between 2015 and 2016 do not show any difference in terms of ROC areas.

**Table 3:** The ROC curves under different areas per category

	Drug		Material		Examination	
	Area	Prob.	Area	Prob.	Area	Prob.
2015	0.654	0.001	0.655	0.001	0.666	0.015
2016	0.648	0.001	0.631	0.005	0.680	0.008

### A dashboard for showing the abnormal items

**Fig. 2:**
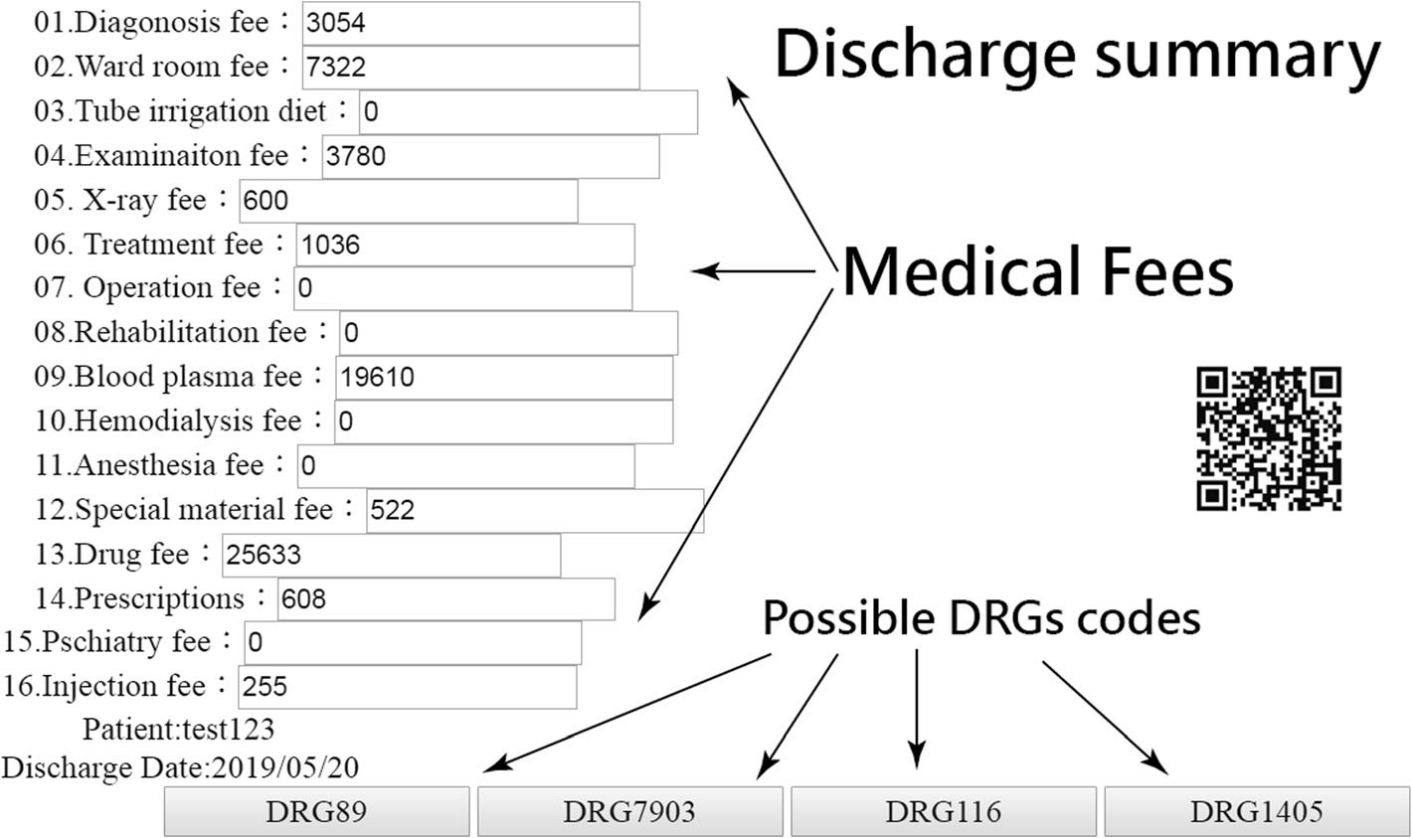
The snapshot from the computer screen after completing discharge summary and before the online DRGs check connected to API on website

**Fig. 3:**
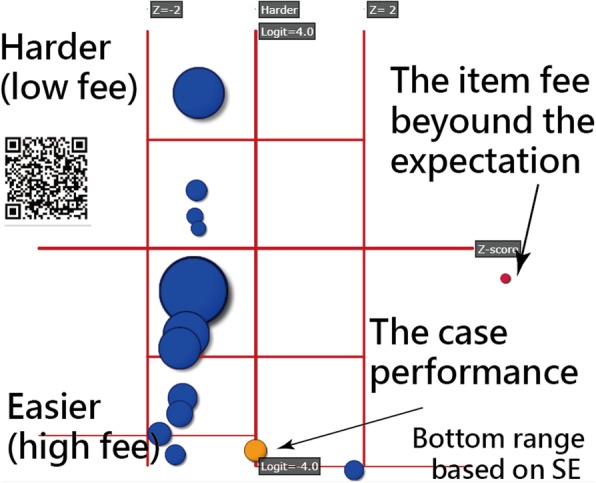
The visual display on a dashboard for the abnormal items

The scenarios were set as one physician finished the patient discharge summary and then clicked on one icon for displaying the possible TW-DRGs classified based on the primary, second diagnoses, and other criteria (e.g., gender, age, complication or comorbidity (CC), etc.). These assigned DRG codes were linked with patient ID, discharge date, and the medical fees, see Fig. 2. The results immediately appear on cloud computing (Fig. 3). The demonstrations for creating hyperlinks on the website were shown in Additional files 1 and 2.

The item difficulties are on the Y-axis. The fee items on one TW-DRG is shown by bubbles on the dashboard, indicating the harder difficulties are on the top. By contrast, the Z-scores are on the X-axis. The appearance on the right-hand side implies that the fee might be beyond our expectation(i..e, too higher than the model standard), with high probability outside the criterion (i.e., > 2.0). The negative Z-score refers to the fee under the model expectation(i..e, too lower than the model standard).

The bubble size represents the standard error of difficulty for an item. We illustrate in Fig. 3 on which the fees of psychiatry presents a more difficult medical expenditure on the TW-DRG shown at the topmost part. The accommodation fee for room, located at the most bottom part, indicates the highest fee among the medical expenditures(i.e., the easiest item on IRT terms). Only the blood plasma fee shown at the right side with a red bubble in Fig. 3 presents the Z-score beyond the criterion (i.e., Z = 3.62 > 2.0) with difficulty = − 0.53 logits, SE = 0.13 and the original 19,610 claimed NT dollars for this inpatient discharged case.

The yellow bubble at the bottom represents the case performance (= − 3.74 as ability in IRT terms). The outfit means square error (MNSQ) for the case is 1.49 (< 2.0), implying that the extent of abnormity is not severe enough as a whole. Interested readers are recommended to scan the QR-code in Fig. 3 to see the details on the dashboard online.

## Discussions

We use the norm-criterion concept on TW-DRGs to examine the outlier items for individual DRG cases using the Z-score(> 2.0), which detects the possible deduction occurs.

Artificially influencing the case mix of hospitals may have several deleterious consequences for the hospital care system [19]. An objective method to distinguish over-evaluation (up-coding) and under-evaluation (under-coding) of the case-mix is required to develop in health-care management settings [20–23].

We also found the following outcomes: (1) using the standardized residuals yielded a moderate accuracy rate [22]; (2) the drug category presents the lowest accuracy (= 0.39 in Table 1) in terms of deduction prediction on reimbursement, thereby indicating that this might be the most crucial conflict point between the BNHI and the hospitals; and (3) the visual display shown on an online dashboard in Fig. 3 can be applied to clinical settings, and help physicians in making adjustments and notes on patient records, which can be used in the preparation of future assessments by the BNHI.

### A tool for DRG assessment

The use of TW-DRGs has been implemented in Taiwan since 2010. Under the assumption that the DRGs with similar fee structures have identical DRG codes, we examined whether the deductions on fees by the BNHI have a significant association with the Z-score estimated by the Rasch model of continuous variables [7, 8]. This approach is much different from the traditional ways applied by the BNHI and randomly assigned to physicians for each case in examining which medical fees in reimbursement should be deducted. The one of cloud computing performed in this study can be helpful to both the BNHI and the hospitals in assessing abnormal medical cases of reimbursements or claims, thus mitigating the occurrence of arguments between both sides in the future.

Many studies [5, 24] have discussed the up-coding behaviors of DRG. That is to inspect whether any feasible and viable monitoring system that can distinguish over-evaluation (up-coding) and under-evaluation (under-coding) of the case-mix. However, no such reliable and practical research had been proposed before. None applies scientific model concepts to deal with the up-coding on DRGs. Particularly, no such software, or say API, that can solve the problem of detecting DRGs up-coding issue.

### Standardized residual analysis

The standardized model residuals used for detecting the abnormal items might be something related to behaviors. The KIDNAP (as in Fig. 3) used in educational and psychometrical fields has been presented in the literature [10, 11, 25]. The feature that is different from the prior research is that the CAT concept is applied to detect the abnormality because of some missing items in an inpatient case, which are often difficult to deal with using the classic test theory.

In hospital settings, we can imagine that a physician can quickly obtain the NKIDNAP (as in Fig. 3) from the online cloud computing once the discharge summary was finished. The possible deduction in medical fees can be predicted by using the online module we developed in this study. With the application of this technique, fee deductions and future arguments with the BNHI in reimbursements can be prevented in the future.

### Norm-reference of TW-DRGs

The modes of TW-DRGs built by our authors can be applied to other TW-DRGs. That is, each TW-DRG should be examined by our module, and each item difficulty should be calibrated ahead of the implementation in detection. The principle of the parameter estimations for the cases and the items follow the item–case pattern. If all modes have been built, any kind of TW-DRG can be applied to the online cloud computing, once the DRG code and the medical fees are assigned to the linkage and then feedback is presented via the KIDNAP (as in Fig. 3). Physicians and programmers can easily apply this technique to the relevant fields and disciplines in practice. Interested readers are invited to scan the QR-codes on Figures to see more information about the KIDNAP plot and practice it in their ways. The Rasch model for continuous item responses can be referred to the website [26] we designed for understanding the features and characteristics.

### Study limitations and suggestions

As we only applied data from one hospital in verifying the accuracy of the detection of effective deduction by the BNHI, the findings cannot be easily generalized to other hospitals due to the different attributes and characteristics of each institution. However, the method employed in this study, such as the comparison of two groups and the inference on cloud computing, is worthy of further investigations in the future.

DRGs with similar clinical characteristics, resource consumption patterns, and comparable costs [2] are the basis of norm-reference. If the assumption is violated, the inference made in this study will be in vain. Hence, online cloud computing should be further examined in future works.

Cautious readers may question that the number of 194 and 300 cases used in this study cannot be considered as big enough to support the inference. We clarify the limitation that used so small sample size for calibrating model parameters. In practice, the Rasch model is not like other IRT models requiring a large sample size to calibrate more parameters in a model. Many studies enrolled a smaller sample size in Rasch analysis, such as 167 [27], 497 [28], and 93 [29].

As for the 194cases in the experimental group used for verifying the effectiveness of the method, we suggest that many cases should be reexamined in the future for ensuring the scientific API viable and useful in clinical settings.

## Conclusion

The DRGs-based comparisons in the possible deductions on medical fees, along with the algorithm on Rasch modeling, have the potential to be applied to other institutes, not just BNHI, on tools for upgrading the efficiency and accuracy in processing medical fee applications in the discernable future.

## Additional files

Additional file 1: Study dataset. (XLSX 1030 kb)

Additional file 2: The demonstration of DRGs detection on website. http://www.healthup.org.tw/kpiall/quest2/drgdetect.htm. (TXT 125 bytes)

Additional file 3: Demonstration. (MP4 985 kb)

Additional file 4: Excel module of the Rasch model for continuous responses. (XLSM 3009 kb)

## Data Availability

The datasets generated and analyzed during the current study are available in the Additional file 1.

## Abbreviations

API: Application programming interface
AUC: Areas under the ROC curve
BNHI: Bureau of National Health Insurance
DRG: Diagnosis-related group
FFS: Fee-for-service
IRT: Item response theory
NHI: National Health Insurance
ROC: Receiver operating characteristic curve
